# Maternal Immune Dysregulation and Autism–Understanding the Role of Cytokines, Chemokines and Autoantibodies

**DOI:** 10.3389/fpsyt.2022.834910

**Published:** 2022-06-02

**Authors:** Janna McLellan, Danielle H. J. Kim, Matthew Bruce, Alexandra Ramirez-Celis, Judy Van de Water

**Affiliations:** ^1^Division of Rheumatology, Department of Internal Medicine, Allergy, and Clinical Immunology, University of California, Davis, Davis, CA, United States; ^2^MIND Institute, University of California, Davis, Davis, CA, United States

**Keywords:** autoantibodies - blood, cytokines, chemokines, neurodevelopment, inflammation, gestational inflammation

## Abstract

Autism spectrum disorder (ASD) is acknowledged as a highly heterogeneous, behaviorally defined neurodevelopmental disorder with multiple etiologies. In addition to its high heritability, we have come to recognize a role for maternal immune system dysregulation as a prominent risk factor for the development of ASD in the child. Examples of these risk factors include altered cytokine/chemokine activity and the presence of autoantibodies in mothers that are reactive to proteins in the developing brain. In addition to large clinical studies, the development of pre-clinical models enables the ability to evaluate the cellular and molecular underpinnings of immune-related pathology. For example, the novel animal models of maternal autoantibody-related (MAR) ASD described herein will serve as a preclinical platform for the future testing of targeted therapeutics for one ‘type’ of ASD. Identification of the cellular targets will advance precision medicine efforts toward tailored therapeutics and prevention. This minireview highlights emerging evidence for the role of maternal immune dysregulation as a potential biomarker, as well as a pathologically relevant mechanism for the development of ASD in offspring. Further, we will discuss the current limitations of these models as well as potential avenues for future research.

## Introduction

Autism spectrum disorder (ASD) is acknowledged as a highly heterogeneous, behaviorally defined neurodevelopmental disorder with multiple etiologies. In addition to its high heritability, we have come to recognize a role for maternal immune system dysregulation as a prominent risk factor for the development of ASD in the child. Examples of these risk factors include altered cytokine/chemokine activity and the presence of autoantibodies in mothers that are reactive to proteins in the developing brain. In addition to large clinical studies, the development of pre-clinical models enables the ability to evaluate the cellular and molecular underpinnings of immune-related pathology. For example, the novel animal models of maternal autoantibody-related (MAR) ASD described herein will serve as a preclinical platform for the future testing of targeted therapeutics for one ‘type’ of ASD. Identification of the cellular targets will advance precision medicine efforts toward tailored therapeutics and prevention. This minireview highlights emerging evidence for the role of maternal immune dysregulation as a potential biomarker, as well as a pathologically relevant mechanism for the development of ASD in offspring. Further, we will discuss the current limitations of these models as well as potential avenues for future research.

## The Role of the Maternal Immune System in the Development of ASD

In the past two decades, an increasingly large number of studies have provided evidence that maternal immune dysregulation can impact offspring neurodevelopment (1–11). For example, it is now well-understood that the maternal immune system plays a critical role in the healthy development of the fetus. In fact, maternal immunoglobulin G (IgG) can cross the placenta and enter the fetal compartment, thereby providing early immunological protection to the infant during the perinatal stage (12, 13). However, this protective mechanism is not selective and harmful antibodies reactive to “self” proteins can also cross the placenta (14). Once within the fetal compartment, these maternal autoantibodies may bind to their protein targets, potentially impacting protein function. This is especially dangerous during the perinatal period given the permissiveness of the blood-brain barrier (BBB) and vulnerability of the developing central nervous system (CNS) (15). In addition to the production of autoantibodies, other perturbations of the maternal immune system can result in harmful effects on the developing fetus. It has been shown that alterations in the maternal cytokine/chemokine profile can result in changes in the inflammatory state, skewed cellular signaling, and altered neurodevelopment (16, 17).

## Cytokines and Chemokines as Potential Biomarkers

In recent years, several researchers have reported on specific cytokines and/or chemokines that are associated with ASD risk both in children with ASD and mothers that have children diagnosed with ASD. Interestingly, several cytokines and chemokines are constitutively expressed throughout the developing CNS (18, 19). Cytokines in the brain perform functions similar to those seen in the periphery, acting as cues in cell development and differentiation, and regulating the types and number of neuronal and non-neuronal cells (20, 21). Together, these complex processes contribute to early neurodevelopment and homeostasis. In the context of maternal-fetal interaction, some cytokines, in particular IL-6, can be transported from the mother to the offspring and access the developing CNS (17). Although the source of abnormal cytokine levels in mothers during gestation may differ, this could alter placental production and overall levels of cytokines and chemokines in the fetal compartment, impacting the cascade of neurodevelopment.

To date, a variety of clinical studies have demonstrated altered cytokine levels in mothers and neonatal blood samples that are strongly associated with ASD (Table 1), increasing the potential for cytokines/chemokines to serve as biomarkers for risk of abnormal neurodevelopment. Most of the reported cytokines associated with ASD induce the BBB to become more permissive (22) increasing the vulnerability and altering the homeostatic levels of cytokines in the developing CNS. Numerous researchers have corroborated the concept that significant changes in maternal cytokine/chemokine homeostasis are correlated with ASD development even in the absence of maternal/fetal infection. Such changes in maternal cytokines and chemokines can not only directly impact the fetus but can also stimulate placental production of pro-inflammatory cytokines within the fetal compartment (17, 23, 24). For example, dysregulation of serum and/or amniotic fluid IL-4, IL-6, and IL-8 have been described in mothers of children with ASD and were associated with altered behavioral outcomes including non-verbal cognitive ability and stereotypical behaviors in children (25–28). The cytokine IL-17, which has been implicated in several neurodevelopmental disorders, including ASD, has been shown to promote inflammation and disrupt the BBB postnatally (29, 30). In addition, IFN-γ, which is involved in neuronal differentiation and synaptic formation, was shown to be higher in mothers of children with ASD during the second trimester (25, 28), potentially suggesting disrupted synaptic connectivity.

**Table 1:** Maternal cytokines/chemokines and maternal autoantibody targets implicated in ASD.

**Cytokines/chemokines**	**Sample source**	**Reference**
IL-1, IL-4, IL-5, IL-8, IL-7A, IFN-γ	Maternal serum collected mid-gestation	(25, 26, 28)
IL-4, IL-10 MCP-1 TNF-α, TNF-β	Amniotic fluid	(27, 32)
Target antigen	**Protein function**	
CRMP1, CRMP2, STIP1, YBOX	Microtubule dynamics, protein chaperone, transcription factor	(12–14, 42–44)
LDH-A, LDH-B, GDA, NSE	Metabolic enzymes	(14–16, 37–41)
CASPR2	Cell adhesion	(45, 52)

Studies have also observed altered cytokine and chemokine levels in neonates who are later diagnosed with autism. For example, researchers found that newborns later diagnosed with ASD have higher peripheral levels of IL-1β, which is expressed throughout the CNS particularly during the early neurodevelopmental period (16, 21). In our recent findings, CCL27, also known as CTACK, was significantly lower in newborns later diagnosed with ASD. This decrease was associated with an increase in the risk of both ASD with and without intellectual disability when compared to newborns with developmental delay or those with typical development (31). Elevated levels of monocyte chemoattractant protein-1 (MCP-1) and macrophage-derived chemokine (MDC) were found in the amniotic fluid, plasma, cerebellum, and cerebrospinal fluid of children with ASD (10, 32, 33), implying involvement of these chemokines in neurodevelopment. Increased levels of the chemokines macrophage inflammatory protein-1 (MIP-1) and eotaxin are also often noted in children with ASD, and have been associated with behavioral deficits (10, 34–36).

It is unlikely that one specific cytokine or chemokine plays a role in the development of ASD, but rather a combination of cytokines/chemokines are more likely to contribute to the neuropathology associated with autism. The exact consequences of altered cytokine/chemokine levels during pregnancy that lead to the ASD phenotype is an important area of research. Furthermore, the trigger(s) for changes in maternal cytokine and chemokine levels, and how other aspects of the maternal immune system may also be affected are essential questions to address. Although the number of research studies that include time-specific/longitudinal examination as well as mother-infant pair-matched studies are lacking, it is promising that similar groups of cytokines and chemokines are frequently shown to be altered in both children with ASD and their mothers, increasing the promise of cytokines and chemokines as biomarkers for risk of altered neurodevelopment.

## Maternal Autoantibody Targets in Fetal Brain

Several groups have now identified multiple brain antigens that cross-react with maternal IgG and are related to neurodevelopmental impairments in the child, including ASD (37–45). Early studies used fetal brain extracts to probe maternal plasma by western blot (WB) and reported reactivity to bands at ~36 kDa that were present in 10% of the ASD samples vs. only 2% of controls. In addition, they observed increased maternal IgG cross-reactivity to multiple bands at 27, 36, at 73 kD in the ASD-positive mothers (46). Concurrent studies reported similar band patterns of reactivity at 37/73 kDa and 39/73 kDa which were ASD specific and not present in controls (47–50). Several groups, including our own, conducted a series of experiments that lead to the identification of eight maternal autoantibody-related (MAR) ASD antigens and their pathogenic epitopes (51–54). The proteins were identified as collapsin response mediator proteins 1 and 2 (CRMP1, CRMP2), guanine deaminase or cypin (GDA), lactate dehydrogenase A and B (LDHA, LDHB), neuron-specific enolase (NSE), stress-induced phosphoprotein-1 (STIP1), and Y-box binding protein 1 (YBX1) (functions are summarized in Table 1). In this early study, instances of maternal seroreactivity to at least one of the proteins in both the case and control groups was observed, indicating that reactivity to any single antigen is not enough to predict ASD risk. Instead, reactivity to a combination of two or more specific antigens (MAR ASD patterns) increased the risk of a child developing ASD with high accuracy: up to 20% of ASD cases and <1% of the controls; the most relevant MAR ASD pattern found was LDHA+LDHB+CRMP1+STIP1 (23% ASD vs. 1% TD) (51). In a more recent study using machine learning techniques, Ramiz-Celis et al. demonstrated that autoantibody combinations composed of CRMP1+CRMP2, CRMP1+GDA, and NSE+STIP1 predicted up to 20% of ASD cases with 100% accuracy, suggesting a significant potential for these patterns as biomarkers for ASD risk (52).

Other groups have also looked at maternal IgG reactivity to individual brain antigens as potential ASD predictors. Due to their pivotal roles in neurodevelopment, the two most well-studied candidates are contactin associated protein 2 (CASPR2) (43, 44, 55) and N- methyl-D-aspartate receptor (NMDA receptor) (56). Further, several groups have reported that maternal seroreactivity to these proteins is associated with neurological alterations in the offspring, including ASD (37, 38, 40, 57, 58). For example, Brimberg et al. reported that anti-CASPR2 antibodies were elevated in mothers of a child with autism when compared to the controls (37% ASD vs. 8% TD) (44, 59). However, results from a recent Danish study that examined maternal IgG cross-reactivity against several brain proteins (including CASPR2 and NMDA) and child outcomes concluded that seroreactivity to both proteins was associated with intellectual disability but not with ASD specifically (43, 60). However, experiments using animal models suggests that maternal autoantibodies against CASPR2 and NMDA can disrupt proper neuronal function/development and result in ASD-like manifestations in the offspring (43–45, 56). Given data from the various studies, the pathologic significance of gestational exposure to maternal autoantibodies is clearly complex and future analyses will be needed to determine how their presence leads to specific changes in neurodevelopment.

## Maternal Autoantibodies as Potential ASD-Risk Biomarkers

In one study of 450 mothers of children with ASD and 342 mothers of TD children, the presence of select autoantibodies in maternal blood was associated with ~20% of ASD cases compared to <1% of the typically developing (TD) controls, suggesting that these autoantibodies appear to be highly specific in their ability to detect risk of a child getting an ASD diagnosis (51, 52). Therefore, at this point in time, the association of autism with maternal autoantibodies to proteins in developing brain is higher than any single gene mutation described thus far (61, 62). This MAR subtype of ASD is now a focus of intense clinical and pre-clinical research. It is believed that the ASD phenotype observed in children of mothers with these autoantibodies is the result of gestational exposure to these pathologically significant autoantibodies (47, 63–68). While rigorous clinical validation is still necessary, maternal autoantibodies have the potential to be used as a precision medicine tool to evaluate the risk of a child being diagnosed with autism. The etiological relevance of MAR ASD was supported by multiple clinical studies in diverse populations and by experimental rodent and non-human primate animal models [reviewed in (37, 40, 58)], that in the future could lead to the development of MAR ASD prophylactic treatments for women at risk.

Although MAR ASD profiles are promising ASD-risk biomarkers for a subtype of autism, substantial analytical and clinical validation is still needed before they can be introduced into clinical practice. Meanwhile, the use of *in vitro* and *in vivo* animal models will allow us to better understand the pathogenic mechanisms of maternal autoantibodies in neurodevelopment. In addition, they will have the potential to facilitate development of prophylactic treatments to mitigate the neurodevelopmental changes associated with MAR ASD.

## Animal Models of MAR ASD

**Figure 1:**
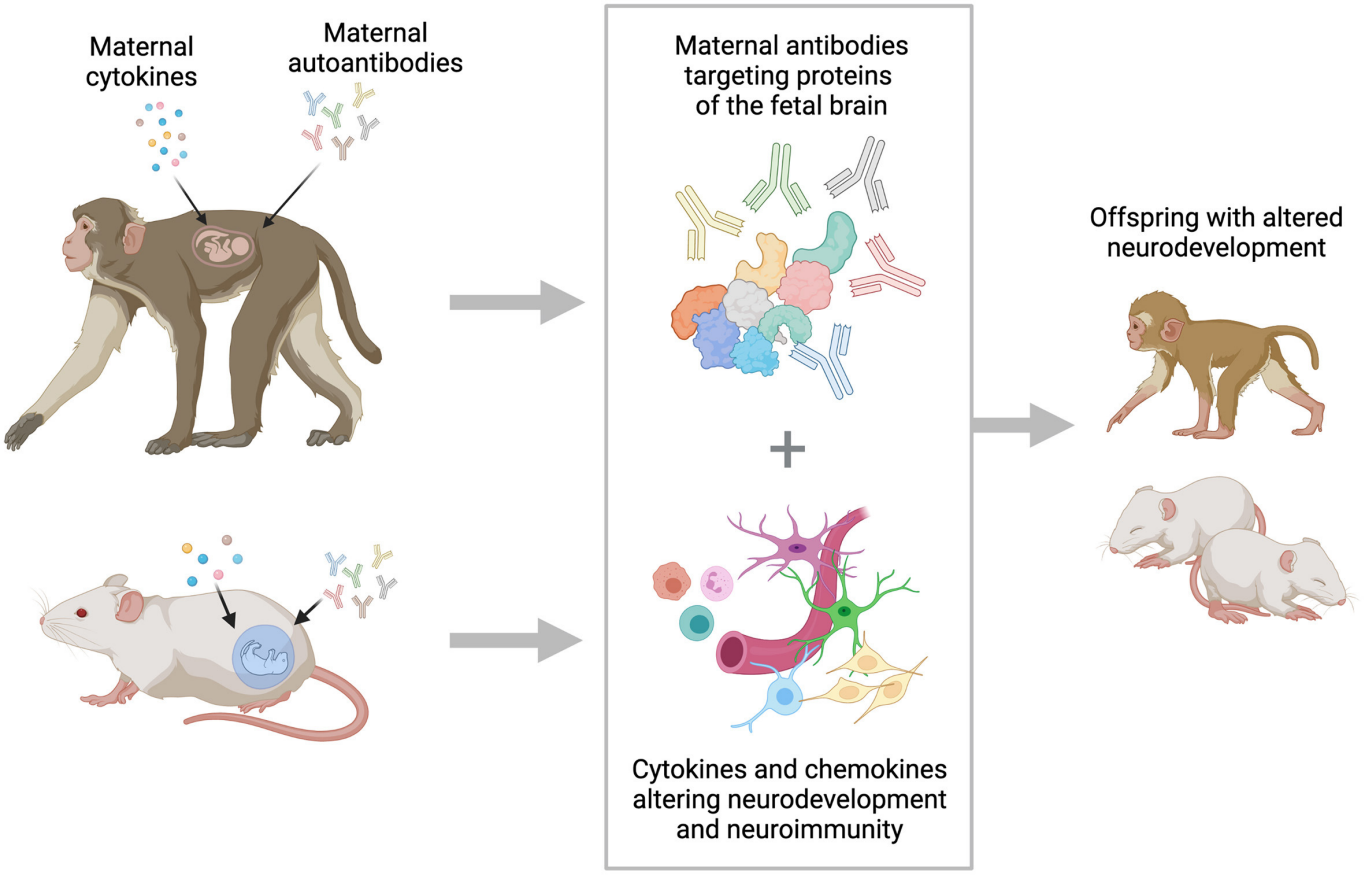
Schematic of maternal cytokines and autoantibodies affecting fetal brain development as studied using animal models. Dysregulation of maternal immune system is a strong risk factor for the development of ASD. Alterations in the production of maternal cytokines and chemokines can impact the neurodevelopmental process, and the presence of maternal autoantibodies reactive to critical proteins can alter how the developmental trajectory of the brain. Pre-clinical models are used to better understand the underlying mechanisms for both proposed pathways to altered neurodevelopment. *The figure was generated by Biorender*.

While strong correlations between the presence of maternal autoantibodies and ASD diagnosis have been observed, the pathological role of maternal autoantibodies in the development of ASD is the focus of ongoing research. Multiple preclinical models, including mice, rats, and non-human primates have been utilized to understand the underlying pathology associated with the presence of these maternal autoantibodies (Figure 1). Early animal models of MAR ASD relied on a passive transfer method, utilizing purified antibodies isolated from mothers of children with autism or from mothers of typically developing children that were transferred to an otherwise healthy animal during gestation. First conducted in mice, and subsequently in non-human primates, the passive transfer models yielded two main findings: (1) They confirmed that maternal antibodies do cross the placenta and can be detected in fetal tissues, including the brain, and (2) they confirmed that exposure to maternal autoantibodies results in changes in offspring behavior and brain development. More specifically, the offspring from mouse dams that received IgG from mothers of children with ASD spent more time in self-grooming and marble-burying behavior, reflective of the stereotypic behaviors observed in ASD. They also showed increased levels of anxiety, a comorbidity many children with ASD experience (69, 70). Furthermore, IgG deposition was observed in the brains of offspring from dams treated with IgG from mothers of children with ASD, but not in controls (69, 70). Similar passive transfer models studying antibodies to CASPR2 and NMDA have also yielded important findings. In one study, mouse offspring exposed to anti-CASPR2 antibodies at gestational day 13.5 had differences in sociability, as determined by the amount of time spent with an unfamiliar object vs. an unfamiliar stimulus mouse. In addition, fetuses examined 2 days after exposure to anti-CASPR2 antibodies showed reduced cortical thickness and changes in cell proliferation (44). Exposure to the NR1 subunit of NMDA receptor at gestational days 13 and 17 resulted in offspring who displayed impaired early postnatal reflexes and decreased prepulse inhibition (56).

In non-human primate passive transfer studies, the results were similar, with offspring from treated dams displaying increased stereotypic behaviors with both novel and familiar social partners (47). When examining the brains of offspring from treated dams, investigators noted that male offspring had a higher rate of brain growth between 3 and 6 months of age, a finding that has been observed in some clinical ASD cases and may be correlated with atypical connectivity and altered brain maturation (71, 72). Together, these studies were essential in validating the hypothesis that maternal autoantibodies have pathologic significance and the potential to alter neurodevelopment (47, 69–71, 73).

The passive transfer model was an essential first step in advancing the understanding of maternal autoantibody pathogenesis. However, this route of administration does not reflect the true exposure a fetus would experience during pregnancy. Thus, to create a more appropriate mimic of the gestational environment where a fetus would be exposed to maternal autoantibodies throughout pregnancy, we generated an antigen-driven model of endogenous exposure. In contrast to the passive transfer model, the antigen-driven model involves immunizing experimental dams with specific protein combinations prior to pregnancy, to induce selective, endogenous autoantibody production (64). Clinical studies had previously identified specific patterns of antigen reactivity as well as the epitopes recognized by the maternal autoantibodies in human samples (51, 53). This knowledge was then used to generate a mouse model with reliable construct validity to clinical MAR ASD. Described briefly, mouse dams were injected with synthetic peptide epitopes for lactate dehydrogenase A and B (LDH-A and B), collapsin response mediator protein 1 (CRMP1), and stress induced phosphoprotein 1 (STIP1), a protein reactivity pattern identified in clinical samples specific to mothers of children with ASD (53). Once immune tolerance to the self-proteins was bypassed, these dams continuously produced autoantibodies to the protein epitopes of interest in the absence of inflammation and other immune perturbations that are also thought to impact offspring development. This approach resulted in offspring that were exposed to the autoantibodies throughout the gestational period, more closely resembling the exposure noted in clinical MAR ASD (64). Using the antigen-driven mouse model, researchers were able to test the behavioral and neurological impacts of constant gestational exposure to maternal autoantibodies. Offspring from treated dams had reduced social behaviors, including fewer bouts of nose-to-nose sniffing and front approaches. In addition, researchers observed elevated repetitive behaviors, decreased ultrasonic vocalizations, and differences in neuroanatomical development; specifically increased brain volume in the offspring from treated dams (64, 74).

Our current studies seek to build upon this foundation through use of an antigen-driven rat model with an expanded repertoire of maternal antibody combinations recently identified in a large clinical study (52). Recent work identifying the consequences of acute peripheral immune activation of offspring neurodevelopment (75), suggests that the rat model is a promising next step in understanding the pathology of maternal autoantibodies. The antigen-driven rat model will allow for a more thorough identification of the behavioral and social impacts of maternal autoantibody exposure, thanks to their more complex neuroanatomy and social interactions. Additionally, utilizing the specific autoantibody combinations identified in clinical research will allow us to identify the consequences of exposure to select autoantibodies, providing researchers with a better understanding of which autoantibody combinations result in more severe pathology, and which can be used as risk-biomarkers. Since the development of this model yields dams that produce autoantibodies in the absence of inflammation, we can directly test how skewing of one arm of the maternal immune system impacts offspring behavior and neuroanatomy. The rat model will not only be more translationally specific, but it will also provide researchers with a pre-clinical model in which to test future therapeutic interventions. In the future, we hope to be able to generate an antigen-driven non-human primate model to further advance our preclinical model.

## Future Directions

Clinical studies of MAR ASD have revealed the critical protein targets for maternal autoantibodies and drawn links between the specific autoantibody combinations and ASD severity. In addition, they have begun to elucidate other immunological factors, such as variances in maternal cytokine/chemokine profile, that can also be used as predictive measures for ASD risk. The use of animal models has helped to confirm the pathologic significance of at least some of the maternal autoantibodies, as well as provided researchers with a means to better understand the downstream molecular consequences and mechanisms involved in these effects. Despite the advances made thus far, many questions remain which, once answered, will aid in the creation of more precise diagnostic tools, preventative treatments, and therapeutics.

There are several questions we are unable to answer using the animal model, such as: What is the triggering event for maternal autoantibody production in humans? What are the longstanding immunological impacts on exposed children? How does previous history of autoimmune disease influence ASD risk through the MAR mechanism? How do autoantibody and cytokine/chemokine levels prior to pregnancy compare to those present during and after pregnancy? To develop targeted therapeutics, it will also be important to further identify when offspring are most vulnerable to autoantibody and/or cytokine/chemokine exposure. To answer these questions, continued clinical studies and use of our pre-clinical models will be necessary. The development of a more sophisticated model using non-human primates would increase the translational ability to the more complex behaviors seen in autism. Although some non-human primate studies have been completed, an antigen-driven model is currently under development, and we remain hopeful that this will soon be another model for researchers to utilize. Future research will continue to elucidate the mechanisms by which maternal autoantibodies impact their target proteins, and the optimal intervention strategies to mitigate damage. In addition, we will aim to identify connections between maternal cytokine/chemokine changes and autoantibody production.

## Conclusions

Autism is an incredibly complex disorder with a wide range of behavioral and cognitive phenotypes. Despite multiple known etiologies, a preventative strategy for the disorder remains to be discovered. Furthermore, testing for specific phenotypic subsets of ASD are also lacking. MAR ASD is one subtype of autism in which the maternal immune system plays a critical role. We now know that specific risk factors exist for immune mediated ASD, including maternal autoantibody production, and skewed maternal and fetal chemokine/cytokine profile. While much is left to be discovered, we continue to use clinical studies and refined animal models to tease apart the mechanisms by which MAR ASD develops. Further, studies are underway to understand how neonatal cytokines and chemokines influence symptom severity as well as the relationship between maternal immune dysregulation during gestation and changes in the brain of affected offspring. We hope this knowledge will yield future preventatives and treatments.

## Author Contributions

This mini-review contains review of research performed by each of the authors. JV is the senior author and oversaw all aspects of this mini-review. JM is the first author and compiled all of the information from the other authors as well as wrote the animal model section. DK wrote the section on maternal immune activation. MB developed the animal models and edited the entire review. AR-C wrote the section on maternal autoantibodies. All authors contributed to the article and approved the submitted version.

## Funding

NICHD funded IDDRC P50 (P50HD103526).

## Conflict of Interest

The authors declare that the research was conducted in the absence of any commercial or financial relationships that could be construed as a potential conflict of interest.

## Publisher's Note

All claims expressed in this article are solely those of the authors and do not necessarily represent those of their affiliated organizations, or those of the publisher, the editors and the reviewers. Any product that may be evaluated in this article, or claim that may be made by its manufacturer, is not guaranteed or endorsed by the publisher.
